# Humeroradial Synostosis: An Updated Classification and Differential Diagnosis Based on Genetic Aetiology

**DOI:** 10.1111/cge.70023

**Published:** 2025-07-17

**Authors:** Fiona Leduc, Clémence Vanlerberghe, Fabienne Escande, Perrine Brunelle, Florence Petit, Anne Dieux

**Affiliations:** ^1^ CHU Lille Université de Lille, Clinique de génétique ‘Guy Fontaine’, ULR7364 RADEME Lille France; ^2^ CHU Lille Université de Lille, Biochimie et Biologie Moléculaire, ULR7364 RADEME Lille France; ^3^ CHU Lille Université de Lille, Institut de Génétique Médicale, ULR7364 RADEME Lille France

**Keywords:** Antley–Bixler syndrome, *CYP26B1*, *DONSON*, humeroradial synostosis, multiple synostoses syndrome, radiohumeral synostosis, *RBM8A*, *WNT7A*

## Abstract

Humeroradial synostosis (HRS) is a rare congenital limb malformation, characterised by fusion of the humeral and radial bones, leading to functional disability of the elbow joint. HRS may be reported in familial or sporadic cases and observed either isolated or as part of a syndromic condition. According to an extensive review of the literature, a dozen known conditions may comprise an HRS. The present review aims to propose an updated classification based on molecular pathways (chondrogenesis and osteogenesis; limb development and patterning; genome regulation), combined with a concise overview of the conditions associated with HRS. This knowledge could guide molecular analyses, patient management and genetic counselling. As some cases remain unexplained, further genetic and epidemiological studies are required to evaluate the contribution of genetic and environmental factors in HRS physiopathology.

## Graphical abstract

The proposed updated classification of humeroradial synostosis is based on the molecular pathways of the genes involved: (1) chondrogenesis and osteogenesis; (2) limb development and patterning; (3) genome regulation. Thus, pathologies belonging to the same molecular type may have overlapping clinical phenotypes, helping to structure the diagnostic approach.

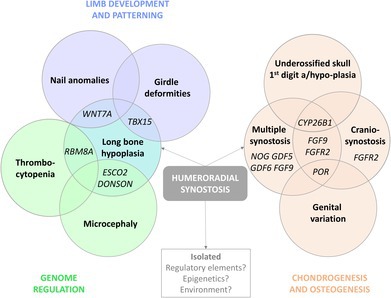

## 1 Introduction

Humeroradial synostosis (HRS) (HP: 0003041), otherwise known as radiohumeral synostosis or fusion, is a rare congenital limb malformation (CLM) of unknown prevalence. It consists of the fusion of the humeral and radial bones, leading to functional disability of the elbow joint. The malformation is either unilateral or bilateral, can occur as an isolated feature or in a syndrome and may be familial or sporadic. Asymmetric involvement results in limb length discrepancy, particularly, if bone hypoplasia is also present. Furthermore, the severity of limb malformations depends on associated bone defects, particularly hypoplasia of the upper limb bones or synostoses. More generally, the prognosis, particularly the cognitive prognosis, varies greatly depending on the identified cause.

The present review proposes a systematic approach to the aetiological diagnosis of HRS. Methods included a review of the literature and relevant databases (Human Phenotype Ontology [1], Online Mendelian Inheritance in Man (OMIM) [2]), together with our accumulated experience as a clinical and biological French expert centre for limb malformations. Descriptions of HRS‐related disorders are based on molecular confirmation in cases where the aetiology is known. The initial assessment is designed to guide subsequent analyses and help variant interpretation according to clinical signs.

## 2 Classification

**FIGURE 1:**
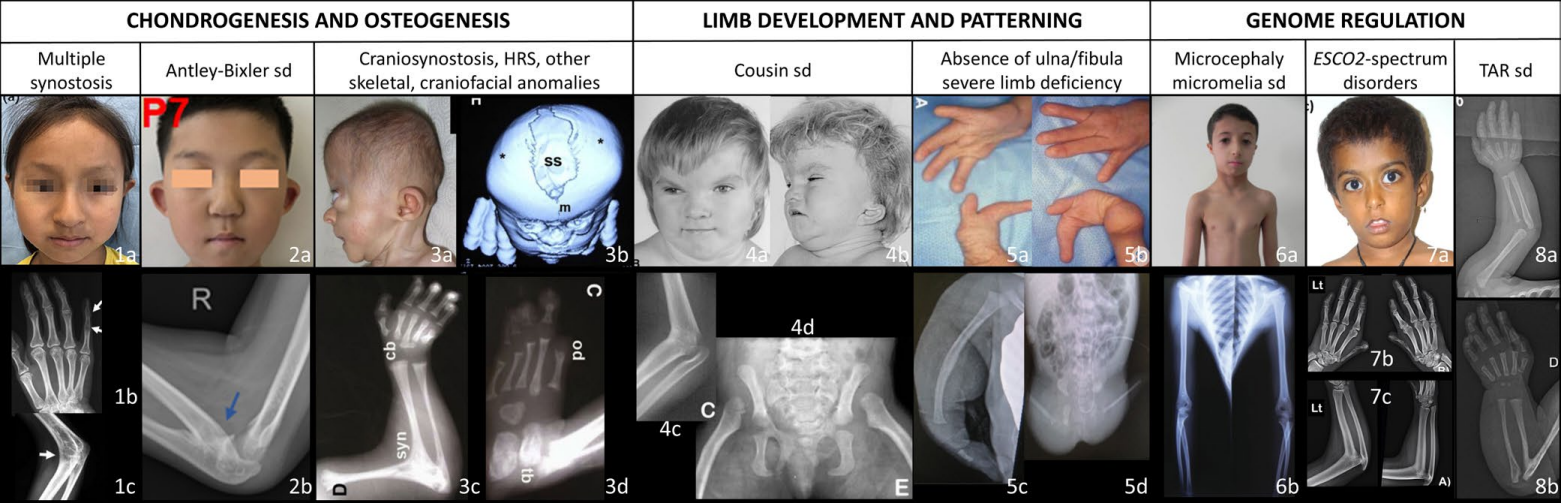
Pictures and x‐rays of patients affected with syndromic humeroradial synostosis, classified according to molecular pathways involved. (1a) Patient carrying a *NOG* variant [4]: note hemicylindrical nose; (1b/1c) patient carrying a *FGF9* variant [5]: note fusions of interphalangeal joints of the fifth digit (1b) and HRS (1c). Patient carrying *POR* biallelic variants [6]: note midface retrusion (2a) and HRS (2b). Patient carrying *CYP26B1* homozygous variant [7]: note midface retrusion and brachycephaly (3a), coronal suture craniosynostosis (3b), HRS (3c), and absence of hallux (3d). Patient carrying *TBX15* homozygous variant [8]: note frontal prominence, narrow palpebral fissures, and hypertelorism (4a), short neck with redundant skin folds at the back and low‐set, posteriorly rotated and dysplastic ears (4b), HRS (4c), iliac bones hypoplasia and dislocation of the femoral head (4d). Patients carrying *WNT7A* homozygous variant [9, 10]: note double‐ventral phenotype with absent nails and postaxial oligodactyly (5a, ventral side of hand; 5b, dorsal side of hand), fusion of arm and forearm long bones (5c), thin femur and pelvic dysplasia (5d). Patients carrying *DONSON* homozygous variant [11]: note beaked nose, microstomia, and micrognathia (6a), hypoplastic long bones (6b). (7a) Patient affected with Roberts syndrome related to *ESCO2* homozygous variant [12]: note exophthalmos and underdeveloped alae nasi; (7b/7c) patient affected with Juberg–Hayward syndrome related to *ESCO2* homozygous variant [13]: note thumb/II/V fingers hypoplasia (7b), and radius hypoplasia and HRS (7c). Patients carrying *RBM8A* biallelic variants [14]: note radius and ulna hypoplasia with thumb presence (8a) and HRS (8b). HRS, humeroradial synostosis; sd, syndrome; TAR, thrombocytopenia‐absent radius. *Source:* Reprinted from Pan et al. [4], Copyright (2020), with permission from Wiley. Reprinted from Laue et al. [7], Copyright (2011), with permission from Elsevier. Reprinted from Lausch et al. [8], Copyright (2008), with permission from Elsevier. Reprinted from AlQattan et al. [9], Copyright (2013), with permission from Elsevier. Reprinted from Boussion et al. [14], Copyright (2020), with permission from Wiley. Reprinted from Schneeberger et al. [12]. Reprinted from Kantaputra et al. [15], Copyright (2020), with permission from Elsevier. Reprinted from Wu et al. [5], Copyright (2020), with permission from Elsevier. Reprinted from Fan et al. [6]. Reprinted from Eyaid et al. [10], Copyright (2011), with permission from Wiley. Reprinted from Karaca et al. [11], Copyright (2019), with permission from Wiley.

The previous classification of elbow synostosis, including HRS, was proposed by MacIntyre and colleagues in 2002 and divided into two classes [3]. Class I synostosis is associated with bone hypoplasia and mostly results in fixed‐extension position. It has been observed mostly in sporadic cases and can be associated with syndromes such as Roberts SC syndrome or femoral fibula ulna complex. Class II synostosis, on the other hand, is characterised by joint maldevelopment and typically results in a fixed‐flexion position. It can be part of multiple synostosis syndrome or Antley–Bixler syndrome and can be familial with autosomal dominant or recessive inheritance [3]. Since MacIntyre et al.'s seminal 2002 paper, no further updates have been published, despite significant advances in genetics analysis and knowledge of the HRS‐related conditions. We propose an updated classification of HRS based on the molecular pathways involved. A summary of the proposed classification, detailing the characteristics of HRS‐associated diseases, is presented in Table 1 and in Figure 1 for iconography.

**TABLE 1:** Proposed classification of the diseases associated with humeroradial synostosis.

Diseases	Genes	Inheritance	Frequence	Occurrence of HRS	Other common upper limb malformation	Common lower limb malformation	Common skeletal features	Specific facial dysmorphism	Common extra‐skeletal signs	
*Chondrogenesis and osteogenesis*										
Multiple synostoses syndrome (MIM #186500, #610017, #612961, #617898)	*NOG, GDF5, FGF9, GDF6* (MIM *602991, *601146, *600921, *601147)	AD	Rare	*NOG, GDF5, FGF9*: frequent (progressive)	Progressive joint synostosis, brachydactyly	Progressive joint synostosis, brachydactyly	Vertebral joint synostosis, pectus carinatum/excavatum; *FGF9*: craniosynostosis (scaphocephaly)	*NOG, GDF5*: hemicylindrical nose, hypoplastic alae nasi; *FGF9*: dolichocephaly	(Progressive conductive hearing loss); *NOG*: hyperopia; *FGF9*: cleft palate; normal cognitive development	
Antley–Bixler syndrome without genital anomalies and disordered steroidogenesis (Type 1) (MIM #207410)	*FGFR2* (MIM *176943)	AD	Extremely rare	Frequent	Multiple joint synostosis, arachnodactyly	Multiple joint synostosis, bowing of femur	Craniosynostosis (brachycephaly, turricephaly), vertebral synostosis	Frontal bossing, midface retrusion, proptosis, depressed nasal bridge, narrow mouth, ear dysplasia	Choanal atresia, hearing loss, hydrocephalus; reduced life expectancy, langage delay, fine motor difficulties	
Antley–Bixler syndrome with genital anomalies and disordered steroidogenesis (Type 2) (MIM # 201750)	*POR* (MIM *124015)	AR	Rare							Genital variations, impaired stereoidogenesis
Pfeiffer syndrome (MIM #101600)	*FGFR2* (MIM *176943)	AD	1:100 000	Occasional	Thumb anomalies	Hallux anomalies	Craniosynostosis (multisuture)	Hypertelorism, midface retrusion, proptosis	Choanal atresia, conductive hearing loss, hydrocephaly, Chiari I malformation, reduced life expectancy, intellectual disability	
Apert syndrome (MIM #101200)	*FGFR2* (MIM *176943)	AD	1–9:100 000	Rare	Progressive multiple joint synostosis, mesoaxial or all‐finger syndactyly, polydactyly	Mesoaxial or all‐finger syndactyly, polydactyly	Craniosynostosis (multisuture including coronal), vertebral fusion	Midface retrusion, proptosis, dental anomalies	Cleft palate, conductive hearing loss, airway obstruction, ventriculomegaly, hydrocephalus, reduced life expectancy, intellectual disability	
Craniosynostosis with radiohumeral fusions and other skeletal and craniofacial anomalies (MIM #614416)	*CYP26B1* (MIM *605207)	AR	12 published patients	5/10	Multiple joint synostosis, camptodactyly, elongated digits, absent/short thumb	Multiple feet joint synostosis, angulated/hypoplasia long bones, elongated toes, absent/short hallux	Delayed ossification/Underossification of skull, craniosynostosis (various type), hypoplastic scapulae, narrow thorax, gracile bones	Downlanting palpebral fissures, midface retrusion, prominent nose, microtia	Spina bifida, encephalocele, hydrocephalus, conductive hearing loss, respiratory failure, possible death in utero or in infancy, intellectual disability	
*Limb development and patterning*										
Cousin syndrome (MIM #260660)	*TBX15* (MIM *604127)	AR	3 published patients	3/3	Brachydactyly	Hypoplastic femur, brachydactyly	Growth retardation, hypoplasia of scapula/ilac bone, abnormal skull	Frontal prominence, narrow palpebral fissures, hypertelorism, short neck with redundant skin folds at the back, low posterior hairline, low‐set, posteriorly rotated and dysplastic ears with narrow auditory canal	(Mixed hearing loss), uncertain cognitive development	
Absence of ulna and fibula with severe limb deficiency (MIM #276820)	*WNT7A* (MIM *601570)	AR	Extremely rare	Frequent	Absence of ulna, postaxial oligodactyly, hypoplasia or absence of nails	Absence of fibula/tibia/toes	No	No	Genital variations, usually normal cognitive development	
*Genome regulation*										
Microcephaly micromelia syndrome (MIM # 251230)	*DONSON* (MIM *611428)	AR	Extremely rare	Frequent	Absent or hypoplastic radius/ulna, oligodactyly involving thumb/fifth finger	Hypoplastic fibula, abnormal hallux, syndactyly, clubfeet	Severe intrauterine and postnatal growth retardation, craniosynostosis, rib anomalies, narrow chest	Short palpebral fissures, broad and beaked nose, microstomia, micrognathia, short neck, low‐set and dysplastic ears	Severe microcephaly, white and grey matter anomalies, absent or hypoplastic corpus callosum, lung hypoplasia, genitourinary malformations, frequent death in utero or in neonate	
Juberg–Hayward syndrome (MIM #216100)	*ESCO2* (MIM *609353)	AR	3 published patients	2/3	Rhizomelic shortening of arms, hypoplastic ulna/radius, fusion of hand bones, brachydactyly/clinodactyly involving thumb/second/fifth fingers	Brachydactyly	Postnatal growth deficiency	Ptosis, hanging nasal columella	Microcephaly, cleft lip/palate, normal cognitive development	
Roberts‐SC phocomelia syndrome (MIM #268300)	*ESCO2* (MIM *609353)	AR	Extremely rare	Rare	Hypo/phocomelia, wrist/elbow synostosis, oligodactly/brachydactyly involving thumb/fifth‐finger	Hypo/phocomelia, knee/ankle synostosis, talipes equinovarus	Intrauterine and postnatal growth deficiency, brachycephaly	Hypertelorism, exophtalmos, downslanted palpebral fissures, malar flattening, underdeveloped alae nasi, micrognathia, ear malformation	Microcephaly, cleft lip/palate, midfacial capillary hemangioma, sparse hair, café‐au‐lait spots, corneal opacity, congenital cardiac/urogenital malformations, possible death in neonate, frequent mild‐to‐severe intellectual disability	
Thrombocytopenia absent radius syndrome (MIM #274000)	*RBM8A* (MIM *605313)	AR	1:100000 to 1:200000	Rare	Absence or hypoplasia of radius/ulna/humerus/shoulder girdles with present thumbs	Phocomelia, hip/patellar dislocations, patella absence, varus/valgus anomalies	Rare (rib and verterbral anomalies)	No	Thrombocytopenia, cow's milk allergy, CAKUT, usually normal cognitive development	
*Clinical entities without known etiological basis*										
Renal dysplasia‐limb defect syndrome (MIM 266910)	Unknown	AR?	5 published patients	2/5	Phocomelia/mesomelia, absence of ulna	Phocomelia/mesomelia, absence of fibula	Rib anomalies	Potter‐like facies	Severe renal hypodysplasia (Potter sequence), genital variations, death after bith	
Femur‐fibula‐ulna syndrome (MIM 228200)	Unknown	Mostly sporadic	Rare	Occasional	Asymetric limb defect involving humerus/ulna, amelia, postaxial finger anomalies	Asymetric limb defect involving femur/fibula, postaxial toe anomalies	No	No	Normal cognitive impairment	
Femoral‐facial syndrome (MIM %134 780)	Unknown (*maternal diabetes*)	Mostly sporadic	Rare	Occasional	No	Asymmetric/unilateral hypoplasia/agenesis of femur, coxa vara, fibula hypoplasia/agenesis, clubfoot	Vertebral anomalies, scoliosis	Mild upslanting palpebral fissures, short nose with broad tip, long philtrum, thin upper lip, microretrognathia	Cleft palate, urogenital anomalies, congenital heart malformation, brain imaging anomalies, motor delay, rare deaths in neonate	
Isolated HRS	Unknown	Sporadic?	Rare	NA	No	No	No	No	No	

Abbreviations: AD, autosomal dominant; AR, autosomal recessive; CAKUT, congenital anomalies of the kidney and urinary tract; HRS, humeroradial synostosis; NA, not applicable.

## 3 Molecular Pathways

For known molecular aetiologies, we divided the HRS classification into three categories according to the molecular pathway of the genes involved: (1) chondrogenesis and osteogenesis, that may correspond to the former Class II; (2) limb development and patterning and (3) genome regulation, both of which may correspond to the former Class I [3]. Thus, pathologies belonging to the same molecular type may have overlapping clinical phenotypes helping to structure the diagnostic approach.

### 3.1 Chondrogenesis and Osteogenesis

#### 3.1.1 Multiple Synostoses Syndrome (*NOG, GDF5, FGF9, GDF6*)

*Noggin* (*NOG*) (MIM *602991), *Growth Differentiation Factor 5* (*GDF5*) (MIM *601146), *Fibroblast Growth Factor 9* (*FGF9*) (MIM *600921) and *GDF6* (MIM *601147) are critical actors involved in joint development by participating in the bone morphogenetic protein (BMP) signaling pathway [16]. These four genes have been associated with multiple synostoses syndromes from 1 to 4 (MIM #186500, #610017, #612961, #617898) [17, 18, 19, 20], all of which are inherited in an autosomal dominant manner.

Multiple synostoses syndrome is characterised by the initial involvement of the extremities, typically the proximal interphalangeal joints and progressively affecting other joints such as the hip, the elbow joints including the humeroradial joint and the vertebral column. The condition is frequently associated with conductive or mixed hearing loss due to stapes fixation or otosclerosis in the case of *GDF6* or *FGF9* variants [19, 20]. Other limb malformations may be observed, including brachydactyly, clinodactyly, syndactyly, radial head dislocation, limited forearm pronation/supination and pectus carinatum/excavatum [4, 17, 18, 19, 20, 21]. Patients carrying *NOG* or *GDF5* pathogenic variants may present with some characteristic nose features [17, 18] (Table 1, Figure 1). Furthermore, patients carrying *FGF9* variants may exhibit craniosynostosis (scaphocephaly, dolichocephaly) and cleft palate [19, 22]. Notably, patients carrying a *GDF6* variant show a different phenotype, characterised by the involvement of carpal and tarsal bones and the vertebral column [16, 20].

#### 3.1.2 Antley–Bixler, Pfeiffer and Apert Syndromes (*FGFR2, FGFR1, POR*)

Antley–Bixler syndrome Types 1 and 2 (MIM # 201750, #207410) are the result of, respectively, a monoallelic variant of the *FGF Receptor 2* (*FGFR2*, MIM *176943) gene or, in more frequent cases, of biallelic variants of the *Cytochrome P450 Oxidoreductase* (*POR*, MIM *124015) gene. NADPH‐cytochrome P450 oxidoreductase is encoded by the *POR* gene and is required for the activity of P450 enzymes (including CYP26B1, see below), which are involved in steroid and hepatic metabolism [23]. FGF ligates to the transmembrane receptors to activate the FGF signaling pathway, which has a wide range of cellular functions including chondrogenesis and osteogenesis in the proximo‐distal limb axis [24].

Antley–Bixler syndrome is characterised, in the first instance, by craniosynostosis (brachycephaly, turricephaly) and HRS. Other skeletal malformations include craniosynostosis‐related facial dysmorphism (Table 1, Figure 1), multiple synostoses affecting the elbow, the extremities and/or the vertebrae, arachnodactyly, camptodactyly, clinodactyly and femoral bowing. Some patients also exhibit neurological anomalies, including hydrocephalus. The patients carrying biallelic *POR* variants may present with genital variations in both sexes and impaired steroidogenesis, predominantly cortisol deficiency. The severity of craniofacial malformations may lead to choanal atresia and early death from respiratory complications. The cognitive phenotype is not well described and seems correlated to the severity of the skull malformations [23, 25]. The previous disease exhibits considerable overlap with Pfeiffer syndrome (MIM #101600) related to *FGFR1* (MIM *136350) and *FGFR2* variants and Apert syndrome (MIM #101200) related to *FGFR2* variants. The two aforementioned conditions are both characterised by multisuture craniosynostosis, craniosynostosis‐related facial dysmorphism, various malformations of the extremities (especially syndactyly in Apert syndrome and first ray anomalies [broad, clinodactyly, brachydactyly] in Pfeiffer syndrome) and brain anomalies, which are frequently associated with intellectual disability. HRS has been occasionally reported in *FGFR2*‐related Pfeiffer and Apert syndromes [26, 27].

#### 3.1.3 Craniosynostosis With Radiohumeral Fusions and Other Skeletal and Craniofacial Anomalies ( *CYP26B1* )

*Cytochrome P450 Subfamily 26B polypeptide 1* (*CYP26B1*, MIM *605207) biallelic variants have been associated with a condition termed ‘Craniosynostosis with radiohumeral fusions and other skeletal and craniofacial anomalies’ (MIM #614416). This disorder manifests with variable severity in the 12 reported patients to date [7, 28, 29, 30]. CYP26B1 is an enzyme catalysing the transformation of retinoic acid, a morphogen playing a significant role in chondrogenesis, into inactive metabolites [7].

The patients exhibit various types of craniosynostosis (coronal, lambdoid, sagittal, multisuture) (6/8) along with delayed ossification of the skull (8/10), hypoplastic scapulae, gracile bones and craniosynostosis‐related facial dysmorphism associated with a prominent nose and microtia (Table 1, Figure 1). CLM comprises multiple synostoses (including HRS: 5/10), absent or short first digits (9/11), elongated digits, camptodactyly and short and bent femurs. The presence of spina bifida, occipital meningocele, hydrocephalus and narrow thorax potentially resulting in respiratory failure is also documented. The spectrum of severity is variable: death may occur in utero (one spontaneous, two by in utero elective termination) or shortly after birth (2/9), the cause of which is not known; survivors exhibit developmental delay and/or intellectual disability (7/7) and conductive hearing impairment [7, 29, 30].

### 3.2 Limb Development and Patterning

#### 3.2.1 Cousin Syndrome (*TBX15*)

T‐Box Transcription Factors (TBX) are a family of proteins that play a crucial role in embryogenesis, particularly in limb development. TBX15 (MIM *604127), which is coexpressed with TBX18 in the limb mesenchyme, has been implicated in skeletal development, mainly in the scapular region [31]. Cousin syndrome (MIM #260660) or pelviscapular dysplasia, is the result of bi‐allelic deleterious variants of the *TBX15* gene and corresponds to a very rare constitutional bone disorder [8, 32]. To date, three unrelated patients carrying homozygous *TBX15* variants have been published.

The clinical presentation is characterised by distinctive dysmorphic features, short stature, hypoplasia of the femurs, scapula and ilia accompanied by dislocation of the hips, mild brachydactyly and an abnormal normocephalic skull. The distinctive dysmorphism is characterised by frontal prominence, narrow palpebral fissures, hypertelorism, low‐set, posteriorly rotated and dysplastic ears with a narrow auditory canal leading to hearing loss, a short neck with redundant skin folds at the back and a low posterior hairline (Figure 1). HRS has been described in all published cases and in our experience (two cases). Notably, no developmental delay was described in the three published patients carrying *TBX15* variants [8, 32]. However, it was reported in the two cases initially described by Cousin et al. [33] without molecular confirmation and it is also present in the two molecularly confirmed cases from our experience.

#### 3.2.2 Absence of Ulna and Fibula With Severe Limb Deficiency (*WNT7A*)

The *WNT family member 7A* (*WNT7A*, MIM *601570) gene encodes a protein that plays a pivotal role in the development of the brain, the female reproductive system and the limbs. Wnt7a is expressed in the dorsal ectoderm of the limb bud and implicated in dorsoventral and anteroposterior polarisation [34]. *WNT7A*‐related disorders, inherited in an autosomal recessive manner [35], are divided into two phenotypes in the OMIM database: (1) Fuhrmann syndrome (MIM #228930), which corresponds to the mild‐clinical spectrum and (2) absence of ulna and fibula with severe limb deficiency (Al Awadi‐Raas‐Rothschild or Schinzel phocomelia syndrome) (MIM #276820), which corresponds to the severe‐clinical spectrum [36]. Genotype–phenotype correlations according to WNT7A residual function have been suggested but are questionable as the same variant has been found in the two syndromes previously described [37].

The disease spectrum is characterised by reduction of the four limbs, with distal segments more affected than proximal, postaxial rays more affected than preaxial and lower limbs more affected than the upper. Absence of ulna and fibula with severe limb deficiency spectrum is typically characterised by absence of the ulna, fibula and tibia and more rarely the femur. These long‐bone defects are associated with postaxial digit reduction, hypoplasia or absence of nails, absence of toes and urogenital anomalies in male and female subjects [35]. Notably, HRS is frequently documented in the severe spectrum [9, 35, 36, 38]. Fusions of lower limb joints were occasionally described [36, 39]. Mild thoracic deformities, characterised by a widened chest cavity, were observed in two unrelated patients with confirmed *WNT7A*‐related disorders from a single case study [9].

### 3.3 Genome Regulation

#### 3.3.1 Thrombocytopenia‐Absent Radius (TAR) Syndrome ( *RBM8A* )

*RNA‐binding motif protein 8A* (*RBM8A*, MIM *605313) encodes a component of the exon junction complex, which plays a regulatory role in mRNA processing. Its haploinsufficiency is responsible for TAR syndrome (MIM #274000) [40] whose estimated prevalence is 1:100 000–1:200 000, with an increase in the African population due to a recurrent hypomorphic variant. TAR syndrome is caused by compound heterozygosity for a null allele (mostly a recurrent 1q21.1 deletion including *RBM8A*) and a hypomorphic *RBM8A* allele, some of which are relatively frequent in the general population or within subpopulations [40, 41].

The disease is typically characterised by absence or hypoplasia of the radius contrasting with preservation of the thumb and neonatal thrombocytopenia. Other common upper limb malformations include hypoplasia or absence of the ulna, humerus and shoulder girdle, syndactyly and fifth‐finger clinodactyly. Furthermore, malformations of the lower limbs, which are generally less severe than those of the upper limbs, include hip and patellar dislocations, absent patella, phocomelia, varus and valgus anomalies. Congenital heart defects, congenital anomalies of the kidney and urinary tract and cow's milk allergy have also been reported as part of the syndrome, while rib and vertebral anomalies have been rarely reported [40, 41]. Although HRS is rarely reported in TAR syndrome, this condition is a diagnosis to consider in HRS based on one previously reported case [14] and our own experience. Furthermore, our experience suggests that some patients may have HRS in the absence of overt radial hypoplasia.

#### 3.3.2 Microcephaly Micromelia Syndrome (*DONSON*)

*Downstream Neighbour of SON* (*DONSON*, MIM *611428) gene, encoding a replisome component ensuring stabilisation of the replication fork, has been linked with two relatively similar autosomal recessive OMIM phenotypes: (1) microcephaly micromelia syndrome (MIM # 251230) [42, 43] and (2) microcephaly, short stature and limb abnormalities (MIM # 617604). The latter is less severe due to a residual DONSON activity [43].

The first description of microcephaly micromelia syndrome was in the Canadian First Nations population of Saskatchewan. This condition is associated with severe intrauterine growth retardation, severe microcephaly and limb malformations. The patients usually die in utero or shortly after birth due to hypoplastic lungs. The limb malformations are severe with delayed bone age, particularly affecting the upper limbs and include oligodactyly (absence of the thumb/fifth finger), absent or hypoplastic radius/ulna and HRS. Distinctive dysmorphic features are observed: short palpebral fissures, broad and beaked nose, microstomia, micrognathia, short neck and low‐set and dysplastic ears (Table 1, Figure 1). Patients may also exhibit lower limb malformations (e.g., clubfeet, hypoplastic fibula/patella, abnormal hallux, syndactyly), craniosynostosis, rib anomalies, narrow chest and genitourinary malformations. Notably, brain imaging reveals significant alterations of white and grey matter, as well as corpus callosum anomalies [42]. Recently, two additional related cases were reported from a different geographical region, exhibiting the same disease spectrum but with more severe manifestations, including anophthalmia, absent feet, scapula and pelvic bone deformities and absent external genitalia [44].

#### 3.3.3 Roberts SC Phocomelia and Juberg–Hayward Syndromes ( *ESCO2* )

Establishment of Sister Chromatid Cohesion N‐Acetyltransferase 2 (ESCO2) (MIM *609353) protein ensures the acetylation of the cohesin complex involved in the cohesion of sister chromatids and gene regulation. Consequently, the karyotype of patients carrying *ESCO2* variants can show a pathognomonic ‘railroad track’ appearance of chromosomes due to repulsion of heterochromatic regions and premature centromere separation. *ESCO2*‐spectrum disorder is inherited in an autosomal recessive manner and includes two phenotypes: (1) Roberts SC phocomelia syndrome (MIM #268300) and (2) Juberg–Hayward syndrome (MIM #216100) [45].

Roberts SC phocomelia syndrome is a rare disorder (approximately 150 published cases including 80 molecularly confirmed) primarily characterised by prenatal and postnatal growth deficiency with variable severity, microcephaly, bilateral hypomelia or phocomelia, with the upper more affected than the lower and specific dysmorphism including hypertelorism, exophthalmos, downslanted palpebral fissures, malar flattening, underdeveloped alae nasi, micrognathia, lip/palate cleft and dysplastic ears (Figure 1). Limb defects are highly variable, from mild thumb defect to phocomelia. Multiple synostoses affecting the wrist, ankle, knee and elbow bones, are also present [45, 46]. It is noteworthy that HRS may be present, although this has been infrequently documented [12]. Other malformations that may be observed include brachydactyly, oligodactyly (the thumb being the first to be affected in frequency following by the fifth finger), clinodactyly/hypoplasia of thumb and fifth finger, ocular features such as corneal opacity, midfacial capillary haemangioma and cardiac and urogenital defects. Associated neurodevelopmental disorders are highly variable. Notably, the severity of limb malformations correlates with the severity of dysmorphic features [45, 46].

Juberg–Hayward syndrome, alternatively termed orocraniodigital syndrome, has historically been characterised by lip/palate cleft, microcephaly, postnatal growth retardation and thumb defects. Recent studies have identified causative biallelic variations of *ESCO2*, thereby confirming the clinical overlap with Roberts SC phocomelia syndrome. A notable observation is the relative sparing of lower limb involvement (possible brachydactyly). HRS is a common sign, as is arm rhizomelic shortening and brachydactyly (involving first the thumbs, the second and the fifth fingers), but only three patients have a molecular confirmation of the diagnosis. Furthermore, there have been no reports of families combining Juberg–Hayward and Roberts SC phocomelia syndromes [13, 15].

### 3.4 Clinical Entities With HRS of Unknown Etiological Basis

Some clinical entities with HRS have been described in the literature without a known molecular cause, making their pathological classification difficult. Based on clinical signs, we differentiate isolated HRS, HRS associated with other limb involvement (see below Sections 3.4.2 and 3.4.3), and syndromic HRS (see Section 3.4.4) (Table 1).

#### 3.4.1 Isolated HRS

Only a few case reports of isolated HRS have been published to date, without any genetic analysis being reported. There have been no reported cases of familial occurrence; however, bilateral HRS suggests the potential for a genetic and/or environmental causative factor rather than a local factor like a vascular defect [47, 48]. The case reports probably reflect past and/or current practice of not performing genetic analyses in these patients, as no genetic cause has been identified in isolated HRS to date. However, before considering HRS as isolated, the evaluation has to be conducted by a medical specialist trained in dysmorphology and genetics with a complete paraclinical evaluation (see Section 4). In the case of isolated HRS, the aetiology could be genetic, either related to a mild clinical spectrum of the above pathologies or to an unidentified molecular mechanism. Alterations of regulatory elements, as described in another CLM [49] or sporadic methylation anomalies could be diagnostic candidates to be explored in research.

#### 3.4.2 Femur–Fibula–Ulna (FFU) Complex

FFU complex (MIM 228200) manifests predominantly in sporadic cases. To date, no genetic or environmental cause underlying this condition has been identified. FFU complex is characterised by asymmetric hypoplasia of the femur, fibula and/or ulna. Other limb malformations include amelia, hypoplasia of the humerus, HRS, bowed tibia and postaxial finger and/or toe defects. The upper limbs are more frequently affected than the lower limbs and the right side is more often affected than the left. It is noteworthy that this description is primarily based on Lenz's paper published in 1992, as only a limited number of case reports were published subsequently [50].

#### 3.4.3 Femoral‐Facial Syndrome (FFS)

FFS (MIM %134 780), also known as femoral hypoplasia‐unusual facies syndrome, is a rare sporadic condition that has been linked to maternal diabetes in approximately 50% of the cases, combining pre‐existing and gestational diabetes [51]. A complex chromosomal rearrangement located in the 2q37 locus has been identified in one case [52].

FFS is characterised by the presence of either bilateral or unilateral femoral hypoplasia/aplasia, which can potentially result in coxa vara and/or hip dislocation as well as motor delay. The ‘unusual facies’ corresponds to retro‐micrognathia with or without cleft palate, long philtrum, thin upper lip and short nose with broad tip. Additional limb malformations may affect the lower limbs, such as fibula hypoplasia/aplasia, clubfeet and preaxial polydactyly. HRS is occasionally seen without other upper limb malformations in these patients. Furthermore, the literature documents the presence of urogenital, vertebral and cardiac malformations, in addition to brain anomalies, including hydrocephalus [51, 53].

#### 3.4.4 Renal Dysplasia‐Limb Defect Syndrome

Renal dysplasia‐limb defect syndrome (MIM 266910), also known as Ulbright‐Hodes syndrome, is an extremely rare condition. To date, there have been four reports published in the literature concerning one sporadic case, a recurrence in siblings and two prenatal cases [54, 55, 56, 57]. This suggests autosomal recessive inheritance, although only one family was consanguineous. The aetiology of the condition remains uncertain since only a karyotype was performed in two cases [54, 56]. It may correspond to a new monogenic syndrome, but an atypical phenotype of a currently known syndrome cannot be excluded.

The condition is characterised by symmetrical limb reduction defects (mesomelia of all limbs, phocomelia of the upper limbs, hypoplastic radius, absence of the fibula and ulna) sometimes accompanied by HRS, rib anomalies, genital variations and severe renal hypo/dysplasia, leading to early death due to Potter sequence [54, 55]. Other signs observed include cerebellar hypoplasia, severe subcutaneous diffuse edema and cleft lip [55, 56].

### 3.5 Associations to Be Confirmed

HRS has been reported in other conditions such as Lenz–Majewski hyperostotic dwarfism (MIM #151050) [58, 59], Rodriguez syndrome (MIM %201170) [60] or in the context of in utero cocaine [61] and methotrexate [62] exposures, but these observations are too rare to establish genotype–phenotype correlations.

## 4 Clinical Practice

**FIGURE 2:**
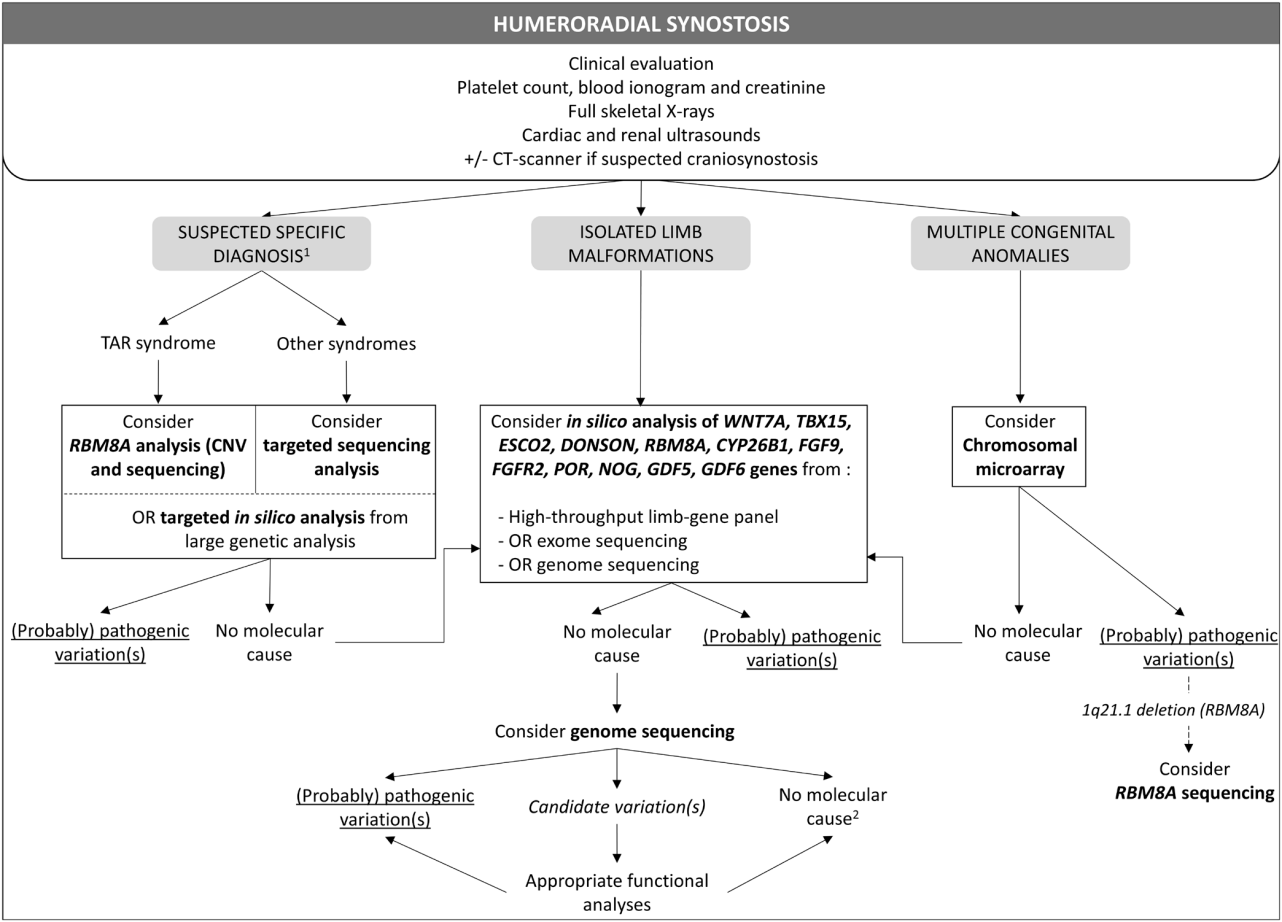
Proposed decision algorithm for investigations in patients with humeroradial synostosis. OR, Note that the choice of genetic analysis may also depend on local clinical practices. (1) See the Table 2 for clinical guidance. (2) Consider a reinterpretation based on new personal or family clinical elements and the evolution of knowledge. Genetic analyses are in bold. TAR, thrombocytopenia‐absent radius.

The preliminary evaluation of HRS‐affected individuals encompasses the collection of pregnancy data, including toxic exposure, pre‐conceptional and gestational diabetes and intrauterine growth retardation. Additionally, it involves the assessment of postnatal growth curves, including height, weight and occipital head circumference, along with the evaluation of psychomotor development. The clinical evaluation has to be conducted by a medical specialist trained in dysmorphology and genetics. This evaluation includes the assessment of facial dysmorphism, which can serve for molecular diagnosis orientation, the search for craniosynostosis, examination of the skin, limbs and joints (Figure 1). Regarding the complementary investigations, we recommend full skeletal x‐rays to identify bone hypoplasia, limb‐girdle and vertebral anomalies. Furthermore, we recommend systematic evaluation of platelet count, blood ionogram and creatinine, as complications may be severe. Finally, we propose the implementation of cardiac and renal ultrasounds to search for malformations and improve preventive care, if deemed necessary. The clinical suspicion of craniosynostosis must be confirmed by CT scan (Figure 2).

Some associated features could orient towards a specific underlying cause: microcephaly (*ESCO2*‐related disorders, microcephaly–micromelia syndrome), craniosynostosis (*FGF*‐related disorders), underossified skull and/or first digit aplasia or hypoplasia (*CYP26B1*‐biallelic variants), girdle deformities (Cousin syndrome), multiple synostosis without craniosynostosis (multiple synostosis syndrome related to *NOG/GDF5/GDF6/FGF9* variant), genital variations (*POR*‐related Antley–Pixler syndrome), thrombocytopenia (TAR syndrome) and nail anomalies (*WNT7A*‐biallelic variants) (Table 2).

**TABLE 2:** Clinical guidance for conditions with humeroradial synostosis.

*Multiple synostosis*	
Craniosynostosis: consider *FGFR2*‐related disorders (Antley–Bixler/Pfeiffer/Apert syndromes) and *FGF9*‐related multiple synostosis syndrome	
	+Underossified skull and/or first digit aplasia or hypoplasia: consider Craniosynostosis with radiohumeral fusions and other skeletal and craniofacial anomalies (*CYP26B1*)
	+Genital variation: consider *POR*‐related Antley–Bixler syndrome
No craniosynostosis: consider multiple synostosis syndrome (*NOG, GDF5, FGF9, GDF6*)	

*Long bone hypoplasia*	
Microcephaly: consider *ESCO2*‐related disorders (Juberg–Hayward/Roberts‐SC phocomelia syndromes) and microcephaly–micromelia syndrome (*DONSON*)	
Thrombocytopenia: consider thrombocytopenia‐absent radius syndrome (*RBM8A*)	
Girdle deformities: consider Cousin syndrome (*TBX15*)	
Nails hypoplasia/aplasia: Consider absence of ulna and fibula with severe limb deficiency (*WNT7A*)	

The diagnosis can also be guided by malformations of the extremities. Thus, proximal interphalangeal fusion is typically observed in multiple synostoses syndrome linked to *NOG, GDF5* and *FGF9* genes, while fusion of carpal and tarsal bones is observed in patients carrying a *GDF6* variant. Absent or short first digit and mesoaxial/all‐fingers syndactyly, associated with craniosynostosis, suggests *CYP26B1*‐related disease and Apert syndrome, respectively. Nails hypoplasia or aplasia, combined with more severe shortening of the lower limbs than the upper limbs, suggests a *WNT7A* variant. Finally, in the case of shortening of the upper limbs, the presence of the thumb suggests TAR syndrome, whereas its absence or hypoplasia suggests microcephaly–micromelia and *ESCO2*‐related disorders.

To our knowledge, no copy number variation has been associated with HRS, with the exception of the heterozygous proximal 1q21.1 deletion including *RBM8A*. Therefore, chromosomal microarray may not have a high diagnostic yield in HRS, except in case of multiple congenital anomalies. Consequently, we propose a molecular analysis encompassing the previously cited genes, such as high‐throughput limb‐gene panel, exome or genome sequencing, with the objectives of enhancing follow‐up management and genetic counselling (Figure 2). It is widely acknowledged that the diagnostic yield is of greater significance in syndromic cases and when CLM is bilateral as opposed to unilateral, though not null in the latter case [63].

## 5 Conclusion

HRS is a rare CLM for which some aetiological factors are known. These can have a significant impact on the follow‐up of patients. Furthermore, a molecular diagnosis allows specifying the recurrence risk in the family depending on the identification of an autosomal dominant or recessive cause associated with HRS. However, in certain instances, such as in cases of FFS and FFU syndrome, no precise etiological diagnosis can be made. The large‐scale implementation of exome and genome sequencing could lead to the identification of novel monogenic causes. Furthermore, isolated HRS may be attributable to alterations in regulatory elements, such as those observed in other isolated CLMs [49]. Genome sequencing should be considered in patients with isolated or syndromic HRS for which no molecular cause has been identified using exome or gene panels sequencing, as it may identify pathogenic or candidate variations (intronic variations, alterations in regulatory elements involved in limb development) (Figure 2). However, the variants interpretation, especially with regard to regulatory elements, must be carried out by biologists specialised in limb malformations and in collaboration with a research team, as the candidate variation must be confirmed by functional analyses.

## Author Contributions

F.L., C.V. and A.D. collected and analyzed the data from the databases. F.L., C.V., F.P. and A.D. designed the work. F.L. wrote the first draft of the manuscript. F.E., P.B., F.P., C.V. and A.D. revised the manuscript. All authors read and approved the final version on the manuscript.

## Ethics Statement

The authors have nothing to report.

## Conflicts of Interest

The authors declare no conflicts of interest.

## Peer Review

The peer review history for this article is available at https://www.webofscience.com/api/gateway/wos/peer‐review/10.1111/cge.70023.

## Data Availability

Data sharing not applicable to this article as no datasets were generated or analysed during the current study.
